# Automated Applications of Acoustics for Stored Product Insect Detection, Monitoring, and Management

**DOI:** 10.3390/insects12030259

**Published:** 2021-03-19

**Authors:** Richard Mankin, David Hagstrum, Min Guo, Panagiotis Eliopoulos, Anastasia Njoroge

**Affiliations:** 1 United States Department of Agriculture, Agricultural Research Service Center for Medical, Agricultural and Veterinary Entomology (CMAVE), Gainesville, FL 32608, USA; 2 Department of Entomology, Kansas State University, Manhattan, KS 66502, USA; hgstr@ksu.edu; 3 School of Computer Science, Shaanxi Normal University, Xi’an 710119, China; guomin@snnu.edu.cn; 4 Department of Agrotechnology, University of Thessaly, 41500 Larissa, Greece; eliopoulos@uth.gr; 5 Tropical Research and Education Center, Institute of Food and Agricultural Sciences, University of Florida, Homestead, FL 33031, USA; anjoroge@ufl.edu

**Keywords:** *Sitophilus oryzae*, *Tribolium castaneum*, abundance, population density, neural networks, machine learning

## Abstract

**Simple Summary:**

A variety of different acoustic devices has been commercialized for detection of hidden insect infestations in stored products, trees, and soil, including a recently introduced device demonstrated in this report to successfully detect rice weevil immatures and adults in grain. Several of the systems have incorporated digital signal processing and statistical analyses such as neural networks and machine learning to distinguish targeted pests from each other and from background noise, enabling automated monitoring of the abundance and distribution of pest insects in stored products, and potentially reducing the need for chemical control. Current and previously available devices are reviewed in the context of the extensive research in stored product insect acoustic detection since 2011. It is expected that further development of acoustic technology for detection and management of stored product insect pests will continue, facilitating automation and decreasing detection and management costs.

**Abstract:**

Acoustic technology provides information difficult to obtain about stored insect behavior, physiology, abundance, and distribution. For example, acoustic detection of immature insects feeding hidden within grain is helpful for accurate monitoring because they can be more abundant than adults and be present in samples without adults. Modern engineering and acoustics have been incorporated into decision support systems for stored product insect management, but with somewhat limited use due to device costs and the skills needed to interpret the data collected. However, inexpensive modern tools may facilitate further incorporation of acoustic technology into the mainstream of pest management and precision agriculture. One such system was tested herein to describe *Sitophilus oryzae* (Coleoptera: Curculionidae) adult and larval movement and feeding in stored grain. Development of improved methods to identify sounds of targeted pest insects, distinguishing them from each other and from background noise, is an active area of current research. The most powerful of the new methods may be machine learning. The methods have different strengths and weaknesses depending on the types of background noise and the signal characteristic of target insect sounds. It is likely that they will facilitate automation of detection and decrease costs of managing stored product insects in the future.

## 1. Introduction

Acoustic technology has a long history of supporting insect pest managers with decision tools and providing contextual information on insect life history, feeding, and movement [1,2,3,4,5,6,7,8,9]. Similar technology is being applied in other areas of agriculture, such as the analysis of cattle behaviors [10,11]. As shown in different sections and tables below, the fundamental and practical utility of acoustic technology has increased over time, due partly to decreases in the costs of sensors and signal recording systems, large increases in signal processing speeds and data storage capability, and rapid expansion in methods to identify sound patterns of targeted insects and discriminate them from background noise. The use of acoustic technology in insect pest management applications increased rapidly between 1980 and 2010 [2] and likely will continue, as global trade and incidental transport of invasive species continue to expand [12,13], heightening the needs for improved detection and monitoring of insects in stored product commodities. The 2011 report [2] covered 137 papers on insect acoustic detection published over more than a century, and this report adds considerably to the total, focusing on the decade since 2010 but including also several relevant papers from previous decades that had not been included in the 2011 report (Table 1).

Table 2 lists applications where acoustic technology has provided information on topics of long-term interest both before and after 2010. In brief, insects have been acoustically monitored not only in stored grain, legumes, fresh fruits, and vegetables, but also in agricultural shipments, bamboo, banana trees, bromeliads, citrus groves, cotton bolls, nursery containers, palm tree groves, turf grass, urban trees, vineyards, and museums containing wooden cultural heritage and musical instruments. Mosquitos have been monitored in salt marshes and trapped to monitor populations of public health interest.

Acoustic monitoring is one of the few methods available for detecting and monitoring the development of hidden larvae feeding internally in stored products, enabling noninvasive documentation of timing when the larvae cease feeding during the molt from one instar to the next (see Section 1.2). Monitoring of feeding reduction and cessation also has been useful in evaluating effectiveness of entomopathogens, host plant resistance, hermetic storage, and insecticides. Insect monitoring studies in Table 2 range from simulated to large scale field tests, and from manual to automated data collection and analysis. Grain damage has been detected in maize, rice, and wheat using impact acoustics. Acoustic trapping of insects also has been used as a monitoring tool. However, the number of studies on detection of infestations is considerably larger than those using acoustics to measure infestation density, possibly because a typically large standard deviation in the rates of sounds produced per insect requires large numbers of samples per study for precise estimations, especially with insects of small size [14,15,16,17,18,19].

The numbers of insect acoustic detection and monitoring studies have greatly increased since 2010 (Table 1) but only a small fraction has dealt with stored product insects. The number of studies on wood borers is nearly as many as with pests in all other studies combined. For the 134 studies cited in Table 2 the average of 6.0 papers per year during the last 10 years is higher than the average for earlier studies from 1988 to 2010 of 3.3 papers per year. Evidently, an overall increased effort to improve acoustic insect detection technology and practical utility has yielded increased interest not only for management of insects in stored products but also for management of insects in other hidden substrates, including trees, interior structures of plants, and underground.

**Table 2:** Examples of different early and recent insect acoustic detection applications.

Research Application	References before 2011	References since 2011
Monitor biology on:		
Feeding, mobility, alarm behavior	[20,21,22,23,24,25,26,27,28,29,30,31,32]	[33,34,35]
Avoidance of oviposition competition in seeds	[36]	[37]
Life history	[24,38]	[39]
Seasonal/daily feeding rates		[40]
Humidity effects on activity/feeding	[41,42,43,44,45,46]	[47,48]
Temperature effects on activity	[49,50]	[8,51,52,53,54,55,56,57]
Temperature preference	[46,58,59]	[60]
Detection/monitoring of:		
Damaged stored food	[61]	[62,63,64]
Stored product insects	[49,65,66,67,68,69]	[70,71,72,73,74,75]
Wood boring insects	[14,76,77,78,79,80,81,82,83,84,85,86,87,88,89,90,91,92,93]	[94,95,96,97,98,99,100,101,102,103,104,105,106,107]
Soil-dwelling & other insects	[108,109,110,111,112,113,114,115,116,117,118,119]	[4,120,121,122,123,124]
Biosecurity and insect density monitoring	[125,126,127,128,129]	[16,17,130,131,132]
Monitoring efficacy of:		
Entomopathogens		[133,134]
Host-plant resistance	[31,135]	
Heat treatment	[136]	[137]
Hermetic and controlled atmosphere storage		[18,74,138]
Insecticide/fumigations	[136,139,140]	[141,142,143]
Trapping of insects to monitor populations	[144,145,146,147,148]	[149,150,151,152]

### 1.1. Improvements over Time in Discrimination/Identification of Insect Sounds

One of the most active areas of insect acoustic detection research is the development of improved methods to identify the sounds of targeted insect species and distinguish them from background noise. Some of these methods are physical, e.g., reduction of background noise by acoustically insulating the samples when feasible ([153,154]). Others involve a variety of feature identification and classification analyses for processing of signals of different characteristics. Table 3 lists some of the most important of the signal preprocessing, feature extraction, and pest identification/discrimination (classification) analyses (e.g., Sharan and Moir [155]) that have been introduced and tested to develop information about pest insect acoustic activity, as well as different statistical methods that have been adapted to distinguish sounds produced by different insect species and different behavioral activities of individual species from each other. The process of feature extraction is termed mathematically as convolution [156]. The automated signal recognition methods in Table 3, (a)–(e) are subdivided into broad categories as follows:

(a) Signal preprocessing. This often includes band-pass filtering of the amplified analog recordings, typically focusing on a frequency band between 80 and 8000 Hz to capture the brief, 3–30 ms impulses produced by insect movement and feeding [2]. The waveform amplitude is a commonly determined signal feature. Many analyses set a threshold visually or statistically and discard signal segments from further analysis if they do not contain above-threshold magnitudes. A commonly applied classification method counts the rate of occurrence of signal segments exceeding the threshold and then classifies stored product samples as infested if the rate exceeds an experimentally determined minimum value. More complex analyses make use of additional signal features such as the total energy or the temporal envelope of short tones or bursts of pulses [157]. Other classification methods employ multiple sensors. Wireless networks employ groups of sensors, spaced either closely together to determine locations of insects in stored grain based on the timing of detection of signal pulses [158], or further apart to estimate widespread populations within a large quantity of stored grain [159] or within multiple trees in an urban area [160].

(b) Neural network classifiers. Artificial neural networks (ANN) are computer models of brain neuron linkage processes that combine weighted inputs from observational data, e.g., acoustic signal pulses, and produce a single binary output that learns its correct value from the observational inputs by use of backpropagation or other methods [161]. Machine learning incorporates neural networks, including convolutional neural networks (CNN) [162], and probabilistic neural networks (PNN), perceptual learning prediction (PLP), decision trees and forests, hidden Markov models (HMM), support vector machines (SVM), Bayesian classifiers, and other methods to improve its predictions automatically through experience with datasets where the target insect species have been independently identified [163,164,165]. Deep learning [156] is a machine learning method that incorporates multiple layers of neural networks, each of which extracts specific features or learned representations of input data [164].

(c) Spectral and temporal pattern features. Specific features of the acoustic signal have been demonstrated to have good predictive value for identifying insects sounds and discriminating them from background noise. Analyses that extract such features include temporal (time domain) pattern identification, spectral, wavelet, and formant analyses, as well as Independent Component Analysis (ICA). In several investigations, acoustic indicators have been constructed that combine information from multiple acoustic features [166,167] or combinations of features from multiple types of sensors [114,168] that provide standardized comparative information useful for targeting insect populations or for monitoring changes in insect and other animal populations in protected areas.

(d) Gaussian Mixture Model (GMM) and Vector Quantization (VQ). These clustering algorithms also are applied to distinguish between features of target signals and background noises [91,169].

(e) Classification algorithms. Subband-based Cepstral Coefficients (SBC) [170], Linear Predictive Cepstral Coefficients (LPCCs) and Mel-Frequency Cepstral Coefficients (MFCCs) [171], as well as wavelets [172,173], KNeighbors [16] classifiers and similar Medium and Complex tree classifications [165] have been used also to distinguish target signals from other signals.

Modern classification methods have different strengths and weaknesses depending on the types of background noises and the characteristics of the insect sounds. Machine learning, including deep learning [174], ultimately may be the most powerful of these methods, provided there is enough training data. The training of convolutional neural networks may require fewer training samples than for deep learning [175]. Similar methods are also becoming widely used in other areas of agriculture [176]. Given initial uncertainty about which classification method to use, researchers may find it most beneficial to begin by first identifying a pivotal question, collecting ground-truth data in relevant environmental contexts, and then testing the success of multiple classification methods before making a final decision about the best signal processing methods to employ.

**Table 3:** Examples of automated signal recognition and classification activities subdivided according to approximate order of first usage with insects (Methods abbreviations are defined in the text).

Method	References
(a) Signal preprocessing, feature extraction,	[125,177,178,179,180,181,182,183,184,185]
Multiple sensors, wireless networks	[129,159,160,163,165,171,186,187,188,189]
(b) neural network classifiers: ANN, machine learning, deep learning, CNN, HMM, SVM, PLP, PNN	[16,48,123,130,162,163,165,171,175,190,191,192,193,194,195,196]
(c) Spectral and temporal pattern features, formant and wavelet analyses	[2,68,87,99,100,101,106,107,197,198,199,200,201,202,203,204,205,206,207]
Time domain signal features, ICA	[48,85,207,208,209]
Polymodal sensor systems, acoustic indicators	[6,168,205,210]
(d) GMM, VQ, denoising, fine gaussian SVM	[91,120,211,212,213,214]
(e) SBC, LPCC, and MFCC analysis, KNeighbors classification	[16,55,56,165,171,213,214,215,216,217,218,219,220]

### 1.2. Advantages of Hidden Immature Insect Detection for Stored Grain Pest Management Decisions

Acoustic technology is particularly valuable for assessing hidden larval infestations in stored grain. Grain samples often have both adult and immature stages of insect pests. Immatures hidden inside the grain kernels are more difficult to separate from grain and identify than adults. The traditional method of detecting immatures is to incubate the grain samples. The incubation time can extend up to two months [221]. To reduce this delay, indirect methods have been proposed based on the determination of obvious indicators of insect presence or activity (mainly focused on advanced larval stages): respiratory gas release, X-ray radiography, flotation, and ninhydrin colorimetric methods [221], as well as acoustic indices of detection [114,166].

Immature insects are often more abundant than adults in seed storages and may be present in samples in which no adults are found. For example, the ratios of immatures to adults in farmer deliveries of wheat to central stores in Australia were estimated at 2.2 for 1962. 4.4 for 1964, 1.1 for 1975, 3.6 for 1977, and 4.4 for 1978 [222]. When the egg stage was omitted because of the difficulty of sampling, X-ray monitoring revealed that larvae, pupae, and adults of *Rhyzopertha dominica* (F.) (Coleoptera Bostrichidae) were 80.6, 10.4, and 9.0% of the population, respectively [126]. These estimates did not differ significantly among samples taken during the 6th, 8th, and 10th week of the study. The 9-fold difference in densities between adult and larval stages was small compared with the 37-fold difference in the number of sounds they produced. In another study that compared ratios of immatures to adults, mean immature to adult ratios were 488.5, 85.9, 71.8, 56.6, 55.8, 17.8, 11.2, 9.7, 5.5, 5.2, 4.4, 3.0, 1.9 in wheat samples from a grain elevator bin [223]. Thirteen locations had immatures but no adults.

In a study of insect infestations in railcars carrying grain, adult *R. dominica* were found in two of eight railcars sampled and represented only 2% of the total lesser grain borer population; whereas, immature *R. dominica* were present in five of the sampled railcars and represented 98% of the total *R. dominica* population [224]. Adult and immature *Cryptolestes. ferrugineus* Stephens (Coleoptera: Laemophloeidae) were found in four railcars and represented 5 and 95% of the *C. ferrugineus* beetle population, respectively [224]. For a growing population, adults represent the previous generations, immature stages are the next generation of adults and the population growth rate will determine immature to adult ratio. Long-lived adults may survive for several generations reducing the immature to adult ratio.

## 2. Materials and Methods

### 2.1. New Technologies since 2011

In the early days of insect acoustic detection research, investigators often had to devise their own sensor systems or modify devices intended for general acoustics [2]. As the field has matured, however, multiple devices have been commercialized to detect insects in stored products, trees, and soil. Table 4 lists several devices with sensors that have been suitable for use with stored product insects, some of which are still in production.

**Table 4:** Recently developed sensor systems applicable to pest insect detection in stored products.

Sensor Label ^1^ (Name)	Device Source	Reference(s)
ec (elec. conduct. sens.)	Research laboratory	[7,225]
ia (impact acoustic)	Research laboratory	[5,61,63,64,226]
lv (laser vibrometer)	Polytec, Berlin, DE	[227]
m (digital stethoscope)	3M Littmann, Maplewood, MN	[228]
m (insect tap)	Research laboratory	[149]
m (red palm weevil detector)	Research laboratory	[229]
mems (micrelectromech.)	Research laboratory	[230]
o (distributed optical fiber, or reflected light)	Research laboratory	[231,232]
o (salient edge detection)	Research laboratory	[233,234]
p (EWD)	Research laboratory	[49]
p (AED 2010L, R15a)	AEC Inc., Fair Oaks, CA, Physical Acoustics, Princeton, NJ	[15,18,34,71,74,75,102,138,235,236,237]
pa (TreeVibes) and related	Insectronics, Chania, Crete, Greece	[17,130,160,238,239]
pa (Postharvest insect Detection System) (PDS)	Cust. Eng. Sol, West Hempstead, NY	[19]
pa	Bosh Sensortech, Reutlingen, GM	[240]
pa (Integr. Circuit Piezo)	Research laboratory	[92,106,241]
pa (electroacoustic sens.)	Research laboratory	[242]
pu (Purdue Biomon.)	Research laboratory	[24,33,135]
pu (focused transducer)	Ultran Labs, Boalsburg, PA	[243]
pvdf (polyvinylidene fluoride)	Piezo & Pyro PVDF & PVDF-TrFE, State Coll, PA	[47]
rm (microwave radar)	Termatrac, Ormeau, QLD, Australia	[56,244,245]
rm (microwave radar)	Research laboratory	[246]
pcr (polymerase chain reaction)	Biotools, Madrid Spain	[247]
ss (seismic sensor)	Agrint, Hod Hasharon, IL	[9]
st (sonic tomography)	PICUS Argus gmbh, Rostock, GM	[101,248]
X-ray		[249]

^1^ ec = electrical conduction, ia = impact acoustic, lv = laser vibrometer, m = microphone, mems = microelectromechanical sensor, o = optical fiber or reflected light, p = contact pickup using PZT piezoelectric transducer, pa = PZT accelerometer (0–20 kHz), pu = PZT ultrasonic transducer (20–200 kHz), pvdf = polyvinylidene fluoride film, rm = resonant microwave radar, ss = seismic sensor, st = sonic tomography.

In subsections below, we consider some of the recent activities in different world areas where local problems have influenced the focus of pest management and parts of the research may have been written in languages other than English.

### 2.2. Methods Development and Application in Europe

Only a few acoustic systems have been evaluated for insect detection in storage facilities in Europe during the last decade. A commonly used and well-studied system has been AED 2010L (Acoust. Emiss. Consult., Fair Oaks, CA, USA), a portable, battery-operated system including a piezoelectric sensor mounted on the end of a metal probe pushed into the grain and a portable acoustic emission amplifier. It has been recently examined in Greece [15,16,17,131,250] and has demonstrated noteworthy efficiency in detecting insect presence inside the grain mass, even in the “critical” density of one or two adult beetles per Kg grain, for a plethora of grain pests such as *Acanthoscelides obtectus* (Say) (Coleoptera: Chrysomelidae) and *Callosobruchus maculatus* (F.) (Coleoptera: Chrysomelidae) [17,131], *Sitophilus oryzae* (L.) (Coleoptera: Curculionidae), *R. dominica*, *Tribolium confusum* Jacquelin du Val (Coleoptera: Tenebrionidae), *Oryzaephilus surinamensis* (L.) (Coleoptera: Silvanidae), *Trogoderma granarium* Everts (Coleoptera: Dermestidae), *C. ferrugineus*, *Lasioderma serricorne* (F.) (Coleoptera: Ptinidae), *Ephestia kuehniella* Zeller (Lepidoptera: Pyralidae), and *Plodia interpunctella* Hübner (Lepidoptera: Pyralidae) [15,16,131,250]. The same system was also successful, under certain conditions, in detecting infestations by dry-woodboring insects like *Hylotrupes bajulus* (L.) (Coleoptera: Cerambycidae), *Anobium punctatum* De Geer (Coleoptera: Ptinidae), *Xestobium rufovillosum* (De Geer) (Coleoptera: Ptinidae), and *Lyctus* sp. (Coleoptera: Bostrichidae) [105] (Table 2).

A different system has been developed and evaluated in Germany, where highly sensitive microphones installed in a metal tube were inserted in the grain mass. The tube not only provided protection for the highly sensitive equipment from dust and other forces but also had acoustical advantages, increasing the surface on which beetle signals could be detected. Additionally, the tube worked as a beetle trap recording all sounds from even one single beetle inside the trap. In lab conditions the infestation could be detected about 8 weeks before a temperature rise, or beetles at the grain surface indicated an infestation [149] (Table 4).

Apart from stored grain pests, bioacoustic methods also have been applied for detection of other cryptic wood-boring insects [97,121,160,239]. For example, detection of the red palm weevil (RPW), *Rhynchophorus ferrugineus* Olivier (Coleoptera: Dryophthoridae), a major pest of various palm species, has been achieved in Israel by a piezoelectric sensor inserted in the fibrous palm tissue. The method was efficient for detecting grown larvae or adults (75–95% sensitivity) but was not successful during the early phase of infestation (33–39% sensitivity) [98]. Similarly, internal larval infestation of banana weevil *Cosmopolites sordidus* (Germar) (Coleoptera: Curculionidae) was acoustically detected (90% accuracy) with a simple sensor (diaphragm stethoscope) connected to a filter to separate the signal from background noises [121].

Acoustic detection methods evaluated in detecting xylophagous insects are very similar to those applied in storage facilities. Acoustic emissions produced by very small wood-boring larvae (1–2 mm length) in wooden objects were measured with piezo-electric sensor and two amplifiers [97]. A novel system called TreeVibes has been recently developed for the automated, real-time, and continuous detection of woodborers inside trees [160,239]. The main parts of the device are an accelerometer and an electronic board that transduces vibrations into audio signals that are stored, compressed and wirelessly transmitted. The TreeVibes demonstrated ~90% detection accuracy in the cases of *Xylotrechus chinensis* (Chevrolat) (Cerambycidae) and *R. ferrugineus*. Many other applications have been suggested for this device, including pest detection in grain silos [160] and grain samples (Section 2.4).

### 2.3. Methods Development and Application in Asia

Initial studies of stored product insect acoustic detection in Asia focused on developing a basic understanding of the magnitude and frequencies of insect-produced signals. Guo and Shang [197,251,252], for example, conducted studies of *Tribolium castaneum* (Herbst) and *O. surinamensis* crawling on film over a microphone. Matlab software (MathWorks, Natick, MA) was used to distinguish the amplitude and frequency of the signals. In a series of reports, Geng, Shang, Bai, Li, and Zhao conducted exploratory studies of crawling sounds detected by microphone from *T. castaneum* adults in wheat, soybean, and corn, and crawling sounds produced in wheat by adults of *S. oryzae* and black fungus beetle, *Alphitobius diaperinus* Panzer (Coleoptera: Tenebrionidae) [154,181,202,204,251,252,253]. Sound spectra of the same species were different in different grains, and the spectra of sounds produced by immatures were different from those of adults. The tests were conducted in an acoustically insulated chamber to reduce extraneous noise. To expand detection to a wider area, Han et al. [159] developed a stored product insect sound detection system connected to a wireless sensor network. Because the ZigBee (Zigbee, Davis, CA, USA) wireless communication system had limited bandwidth, data compression, transmission, and recovery systems were included in the software. Several reviews have been conducted that focused on research progress associated with stored product insect detection technology, including [254,255,256,257,258,259,260,261,262,263,264].

A major focus of stored product insect acoustic detection research in Asia has been in development of methods to discriminate pest insect signals from background noise and other, nontargeted insects. An early approach was work by Guo and Zhang [213] that identified movement sounds of *S. zeamais* (Motschulsky) (Coleoptera: Curculionidae) and *T. castaneum* adults using Mel Frequency Cepstrum coefficients, Gaussian Mixture Models, and clustering. Zhang et al. [172] used wavelet analyses to distinguish *S. oryzae* larvae from *S. zeamais* in wheat, maize and *Coix lacryma-jobi* (Poales Poaceae) millet. The sounds of the stored grain insects depended on the grain size and temperature [265].

Other studies in Asia and Europe have considered physical factors affecting insect acoustic detectability such as the speed of sound and signal attenuation in grain. Guo, Geng, Li, Yu, and others [183,198,265,266,267,268,269] conducted studies to estimate times of sound travel in grain and signal attenuation in grain. These factors also are affected by temperature and moisture [270,271,272,273].

### 2.4. Methods Development and Application in Africa and the Americas

As in Europe (see Section 2.2), the AED 2010L system has been used in Kenya and the US to detect stored product insects in several studies since 2012. Acoustic methods provide early warning of hidden insect presence before devastating infestation thresholds are reached. However, stored product insect infestations in African farmer stores or large grain warehouses are mainly controlled by fumigation and contact insecticide applications, as the usage of detection and monitoring tools is still in the early stages of development [236]. Studies were conducted between 2014 and 2017 in Kenya to assist the development of acoustic sensors for monitoring infestations in food grain warehouses [18,75,236,237]. The research involved lab- and field-based approaches using the AED-2010L (Table 4) as well as older condenser microphone technologies to determine detection capabilities and assemble acoustic profiles [2] of sounds produced by immature and adult stored grain insects, with and without interference from background noise. Acoustic profiles were created for *Prostephanus truncatus,* Horn) (Coleoptera: Bostrichidae), *S. zeamais* (Motschulsky) (Coleoptera: Curculionidae) and *T. castaneum* in maize and *A. obtectus* in beans. The profile ranges of greatest spectral energy were established to be between 3–8 kHz. Acoustic data combined with pit-fall trap-capture data and background noise collected under field conditions in large grain storage facilities provides sufficient information to develop automated decision-support tools [237].

Acoustic detection was also explored for application among organic grain growers in the United States. The project goal was to help organic growers in the US Midwest seeking chemical-free pest management tools for specialty crop storage. Research was conducted in Indiana between 2017 to 2019 on the use of acoustic detection methods as a monitoring tool during hermetic and controlled atmosphere storage of cowpea and wheat [18,75,138]. Laboratory experiments were performed to assess the effect of low oxygen environments on the insect behavior, life cycle, fecundity, susceptibility and resurgence of *S. oryzae* on wheat and *C. maculatus* on cowpea. Low oxygen levels were attained by hermetic sealing of infested grain or purging of infested grain with N_2_. Hermetic sealing reduced the insect acoustic activity below minimum activity threshold levels, <0.02 sound bursts/s, within 14 d while low oxygen levels of 1%, 3%, and 5%, ended insect acoustic activity within 4 d. Ultimately, this research seeks to implement an acoustic monitoring system for testing efficacy of N_2_ (or other gases such as CO_2_) fumigation in grain storage facilities in the Midwest United States.

Besides the AED 2010L, two other acoustic systems have been tested recently in the US to detect stored product insect pests. The Postharvest insect Detection System, PDS, developed by Custom Engineered. Solutions, Inc. (Jeffersonville, Indiana) (Table 4), inputs signals from electret microphones to a 32-bit microcontroller platform that digitizes the sounds and saves them on a memory card for further analyses, enabling discrimination of target insect sounds from background noise and nontarget pest species, as well as population density estimation [19]. The microphones are embedded in a plastic base onto which bags of grain samples are temporarily placed to estimate infestation likelihood.

The capabilities of a second new device, TreeVibes [160,239], were tested for this report. The TreeVibes was connected to a metal probe inserted into three sets of four glass jars, each containing approximately 300 mL of wheat grains artificially infested with 0 (control)–50 *S. oryzae* adults, or with larvae from eggs laid by 0–50 adults in grain over a 1-week period. All insects in the study were reared and maintained as in Mankin et al. [19] and were tested only once. Except for the brief <1 h recording periods, the jars were kept in a rearing chamber at 25.1 ± 1 °C and 50 ± 5% relative humidity. The signals detected by the probes were transmitted from the TreeVibes headphone output to a digital audio recorder (Model PMD661, Marantz, Mahwah, NJ, USA) and saved in “.wav”-file format at a 44.1 kHz digitization rate. To accommodate the limited, 1–8000 Hz frequency range of the accelerometer [160], the signal was bandpass filtered between 160 Hz and 8000 Hz, and then displayed using Raven 1.5 software [274].

## 3. Results

**Figure 1:**
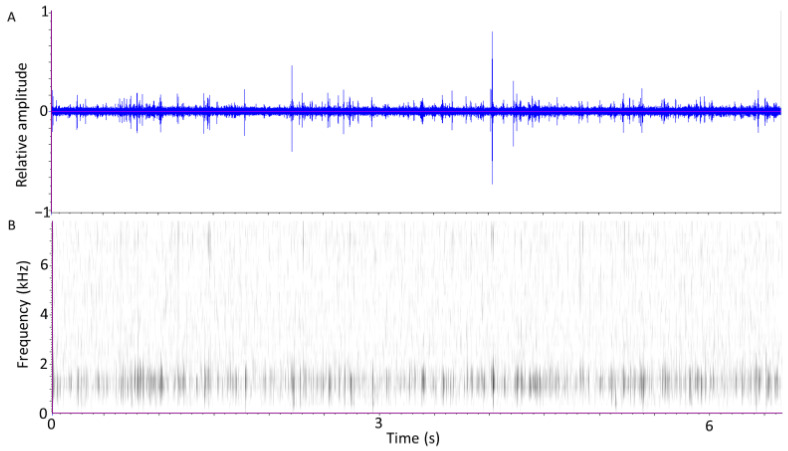
(**A**) Oscillogram and (**B**) Spectrogram of a 6.7 s interval of sounds recorded from 10 adult *S. oryzae*. Darker areas in the spectrogram (calculated at 128 points/spectrum) indicate greater energy at specific frequencies and times.

The TreeVibes device easily detected insect-produced sounds from the 300-mL grain samples infested with either adult or larval *S. oryzae*. Figure 1 displays a 6.7 s period of movement and feeding signals obtained from 10 *S. oryzae* adults that had been placed into a rearing jar filled with wheat grains 6 days previously. The oscillogram (A) displays signal pulses of varying amplitudes spaced in trains (bursts) of varying durations [2]. The spectrogram (B) indicates that the pulses produced by adults typically have their greatest energy between 500 and 1500 Hz, with a few pulses also containing moderate energy between 6–8 kHz.

**Figure 2:**
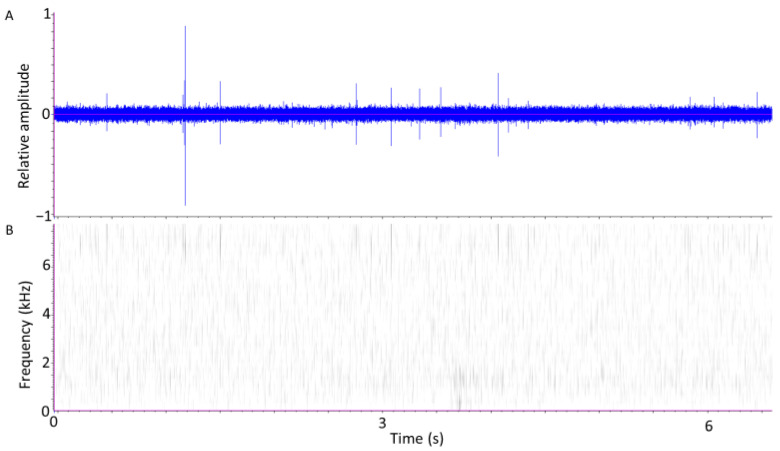
(**A**) Oscillogram and (**B**) Spectrogram of a 6.7 s interval of feeding sounds recorded from *S. oryzae* larvae. Darker areas in the spectrogram (calculated at 128 points/spectrum) indicate greater energy at specific frequencies and times.

Figure 2 displays signals obtained from *S. oryzae* larvae two weeks after 10 adults had been removed from a jar in which they had been introduced for 1 week. Here, the signals are primarily feeding sounds of the larvae hidden within the kernels. As in Figure 1, the oscillogram (A) displays bursts of pulses of varying amplitudes. The spectrogram (B) indicates that the larval pulses have less energy below 2 kHz than the adults and have the greatest energies between 6 and 8 kHz. The total number of larvae was not known in this case but about 300 adults were collected from this test jar over the subsequent three weeks.

**Figure 3:**
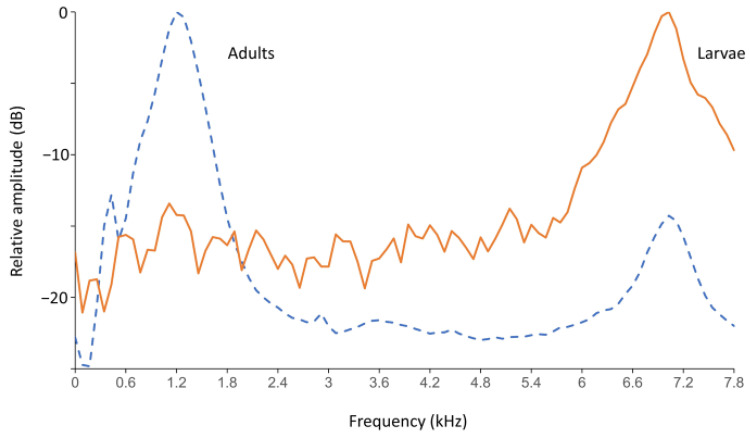
Mean spectral profiles: 10 *Sitophilus oryzae* adults on grain kernels (dashed line) and larvae figure 180 s recordings.

Examples of mean spectra (profiles) of adult and larval sound pulses (Figure 3) were obtained using a custom-written insect sound analysis program, DAVIS [2]. For the adult profile, spectra from 1410 pulses obtained over a 180 s period were averaged from a recording of 10 *S. oryzae* adults placed into a rearing jar 6 days previously. The larval profile was a spectral average of 84 pulses collected by the TreeVibes sensor over 180 s in a jar where 10 adults had been introduced three weeks previously and then removed 1 week later. The shape of the adult profile confirmed results from previous analyses of adult *S. oryzae* sound impulses detected using the PDS device [19], and the larval profile confirmed results previously obtained by Kiobia et al. [71] with *S. oryzae* larvae using an AED 2010L system. In tests with uninfested samples, a few isolated grain-settling sound pulses were detected at rates at or below 0.02 s^−1^, as had been observed also with the PDS device [19]. 

## 4. Discussion

The results of the TreeVibes tests with *S. oryzae* adults and larvae above, as well as those with the PDS device (19), confirm their potential for use in future applications of stored product insect acoustic detection and monitoring. Indeed, as discussed in the subsections below, there is an expanding body of insect acoustic signal detection and analysis studies suggesting that such research will be of growing importance for stored product insect management over the next decade.

It is worthwhile noting that, apart from what has already been described, there are other reviews, research, and theses of relevance to management of stored product insects which do not directly address stored product applications. Such studies include on termites and other tree-boring insects that produce feeding sounds that have spectral and temporal patterns like those of stored product insects [94,95,142,143,235]. Others describe effects of temperature on rates of activity [60]. Others incorporate optical sensors in traps [150,275] with similarities to pitfall traps used for monitoring stored product insects [236]. Finally, we note the increased usage of acoustic detection methods against invasive stored product pests, e.g., *Trogoderma inclusum* LeConte [276] and other invasive *Trogoderma* spp. [15,277].

A recent search for master and Ph.D. theses investigating insect acoustic detection and monitoring found four M.S. and ten Ph.D. theses, seven of which have been published since 2011, (Table 5). They cover a range of topics, from sound propagation in stored grain, to feeding behavior, sound feature extraction, discrimination of insect sounds from noise, methods development, and the use of wireless to obtain insect sounds from multiple locations in storage facilities.

**Table 5:** Recent theses on insect acoustic detection and monitoring.

Author; Type	Year	University	Title
El-Hadad, A.; Ph. D. [101]	2017	U. Melbourne	Using acoustic emission technique with Matlab analysis to detect termites in timber-in-service
Farr, I.; Ph.D. [278]	2007	Univ. York, U.K.	Automated bioacoustic identification of statutory quarantined insect pests
Geng, S.; Ph.D. [279]	2005	Shaanxi Normal Univ., China	Sound characteristics detection, analysis and database construction of stored grain pests
Guo, M.; Ph.D. [267]	2003	Shaanxi Normal Univ.	Propagation of sound signals in quasi-porous media and analysis of the sound properties of pests
Guo, X.; Ph.D. [187]	2007	Zhejiang Univ.	Study on wireless networked control system based on wireless sensor networks
Kiobia, D.O.; M.S. [280]	2015	Virginia Polytech. Inst.	Design and development of a low-cost acoustic device to detect pest infestation in stored maize
Klaassen, R.E; M.S. [281]	1989	Purdue Univ.	Identification of concealed insect infestations using a passive ultrasound monitor
Njoroge, A.W.; Ph.D. [282]	2017	Univ. Kassel	Acoustic detection of insect pests of stored grains in Kenya
Pesho, G.R.; M.S. [283]	1954	Kansas State Col.	Detection of immature rice weevils, *Sitophilus oryzae* L. (Curculionidae, Coleoptera), by audio amplification
Rigato, F.E.; M.S. [284]	2013	Univ Padua	Indagini bioacustiche per l’identificazione di larve di Coleotteri Cerambicidi (Coleoptera Cerambycidae)
Schofield, J.; Ph.D. [285]	2011	Univ. York	Real-time acoustic identification of invasive wood-boring beetles
Watanabe, H.; Ph.D. [35]	2018	Kyoto Univ.	Nondestructive evaluation of larval development and feeding behavior of the bamboo powderpost beetle *Dinoderus minutus* in bamboo culms
Welp, H., Ph.D. [69]	1994	Humbolt-Universitat	Acoustic detection of hidden larvae of several storage pests in products from bioshops of Berlin
Yanase, Y.; Ph.D. [47]	2013	Kyoto Univ.	Development of acoustic emission and gas monitoring methods for nondestructive detection of termite attack on wooden structures

Acoustic technology provides detection of adult and hidden immature insects feeding in grain [59], as well as non-destructive detection of adults and immatures in storage bags, museum collections, aquatic habitats [286], and heritage buildings [57]. The information it provides is electronically transferable to private and public databases for use by researchers, research data alliances, and plant, animal, and human health protection and regulatory agencies [287]. Additional contributions to such databases will benefit future automation of analyses to monitor worldwide abundance, distribution, and diversity of insects [174]. In addition to its increasing usage noted in this report for detection and management of insect pests in stored grain, acoustic technology as well as relevant technology using other sensors is also of considerable interest for management of hidden pest insects underground and in trees, where other detection options may be destructive. All three of these acoustic detection applications enable automated insect pest detection, which will become more important as human populations increase and natural resources decrease. Until now, however, commercial insect detection devices have not yet fully achieved the versatility, ease of use, and cost-effectiveness of currently available cell phones, and are not rapidly supplanting pheromone or probe trap, sifting or other pest insect detection methods in general use. The potential of increased scalability and increased automation nevertheless continues to drive interest from commercial suppliers of insect acoustic detection technology.

Farmers and warehouse managers need information for decision support [288], not only at small sample and large international scales, but also at intermediate scales, which requires both short- and long-term monitoring [289]. Grain samples may need to be tested multiple times at many locations, and the information needs to be relevant to decisions made in local, regional, and international context. Frequently used and transported acoustic detection devices should be both highly sensitive and robust, which increases the costs of production and decreases usage unless the information provided contributes significantly to profits. It has been difficult to reduce costs and automate the interpretation of acoustic signals so that they provide inexpensive, relevant information to end users. Until now, it has been difficult to commercialize devices with such features. Additional research can provide lower-cost devices, greater user friendliness, and greater ease of transfer of relevant information to private and/or public databases. However, the ultimate solution to this conundrum may be to combine acoustic technology with other precision agriculture technologies [290], contributing to automated predictive models and adding value by enabling customization of management treatments at increasingly smaller scales. Some of these technologies already exist at small scale, such as FarmBot (San Luis Obispo, CA, USA).

## 5. Conclusions

For this report, we tested a new device to detect adult and immature insects in stored grain and reviewed the history of different applications of acoustic technology to management of stored product insect pests, described recent advances in automated methods of discriminating insect sounds from background noise as well as recently developed methods for analyzing sounds to distinguish different insects from each other, and reviewed the development of sensor systems applicable to detection of stored product insect pests. In addition, we presented short descriptions of research that has been conducted in recent years on stored product insect management in Europe, Asia, the US, Africa, and elsewhere. After a century of slow development of applications, the use of acoustic methods and automated analyses of pest insect sounds is beginning to develop rapidly, and automated monitoring and management of stored product insect pests is closer to becoming a reality.

## Figures and Tables

**Table 1:** Increases over decades in reports describing insect acoustic detection applications.

Decade	No. Papers Listed	
	Herein	Mankin et al. [2]
1901–1910		1
1911–1920		1
1921–1930		2
1930–1940		4
1941–1950		0
1951–1960		5
1961–1970	1	4
1971–1980	2	4
1981–1990	6	22
1991–2000	14	44
2001–2010	46	50
2011–2020	133	
Total	202	137

## Data Availability

The data presented in this study are available on request from the corresponding author.
